# Rethinking fitspiration in social media: Experimental insights from young Chinese women

**DOI:** 10.1371/journal.pone.0357611

**Published:** 2026-09-03

**Authors:** Siyi Song, Jinfeng Du, Huangkun Wei, Muhamad Shamsul Ibrahim, Hamedi Mohd Adnan

**Affiliations:** 1 Jiangxi University of Software Professional Technology, Nanchang, Jiangxi, China; 2 Department of Media and Communication Studies, Faculty of Arts and Social Sciences, Universiti Malaya, Kuala Lumpur, Malaysia; 3 School of Foreign Languages, South China University of Technology, Guangzhou, Guangdong, China; 4 Jiangxi Institute of Technology, Nanchang, Jiangxi, China; Universiti Malaya, MALAYSIA

## Abstract

As social media platforms increasingly influence contemporary understandings of health and the body, fitspiration—a blend of “fitness” and “inspiration”—has become a widespread visual genre that promotes exercise, discipline, and culturally idealized physiques. Recent research has linked fitspiration content to negative body image among women, yet most studies focus on Western populations. Cultural differences in beauty ideals suggest that findings from Western contexts may not fully apply to Asian women. However, research on fitspiration in Asia, particularly China, remains limited. To address this gap, this study conducted an online experiment with 414 Chinese women aged 18–26, randomly assigned to view thinspiration, fitspiration, or neutral scenery images. Body appreciation and functionality appreciation were measured before and after exposure. Results showed that fitspiration images significantly reduced both body and functionality appreciation compared to neutral images. Thinspiration images produced even lower body and functionality appreciation than fitspiration. This study underscores the need to recognize and critically examine the potential risks of fitspiration content within the Chinese cultural context.

## Introduction

### Social media and negative body image

In recent years, numerous studies have argued that social media use reinforces negative body image perceptions among women [1]. One of the most influential factors is the pervasive presence of ideal body images on social media [2]. A striking example is “thinspiration” (a combination of “thin” and “inspiration”), which refers to images, quotes, and content intended to inspire extreme thinness [3]. According to sociocultural theory, repeated exposure to idealized images may cause individuals to internalize unattainable beauty standards and pursue them [4,5]. This process often leads to upward appearance-based comparisons, thereby exacerbating self-body dissatisfaction [6,7]. Moreover, the effect is particularly pronounced when women experience a discrepancy between their own appearance and the ideal but are unable to close that gap, leading to a decline in well-being, mental health issues (such as depression and anxiety), excessive dieting, and eating disorders [8,9]. These negative impacts are especially severe among young women who frequently use social media and have higher appearance-related expectations [10].

### Fitspiration

To address these issues, various body positive approaches were applied to promote body appreciation, which has been recognized as an effective way to protect against exposure to idealized media content [11]. However, the most represented ways of promoting positive body image in the media (e.g., body diversity) have been criticized to still emphasizing on appearances, with the ignorance of body capabilities [12,13]. Body functionality theory suggests that focusing on “what body can do” rather than “how it looks” can increase body and functionality appreciation [14]. In response, “fitspiration,” a combination of “fit” and “inspiration,” was initially regarded as a healthier alternative to “thinspiration” [11,15]. The movement seeks to encourage individuals to enhance their bodies through healthy lifestyle behaviors such as exercise and healthy eating [15]. Typical fitspiration images depict individuals dressed in athletic attire, posing or engaging in exercise in various contexts, often accompanied by motivational text or content related to healthy eating [15]. Theoretically, fitspiration has the potential to promote a more positive body image [16]. According to the Developmental Theory of Embodiment (DTE), physical experience plays a key role in shaping body image, and a supportive environment for sports or physical health care can encourage healthier living, or lifestyles [17–19]. From this perspective, fitspiration’s emphasis on physical activity and healthy living holds the potential to foster a more positive body image.

However, content analyses of fitspiration images have raised concerns regarding their impact on women’s body image. While fitspiration emphasizes fitness and health, it is still predominantly focused on displaying “thin” bodies [20]. Additionally, the ultimate goal of fitspiration’s promotion of exercise and healthy eating is often to enhance appearance rather than health itself [21]. Furthermore, fitspiration images frequently portray sexualized representations of women, such as images featuring women in revealing yoga outfits highlighting their physical features [22]. Therefore, although fitspiration appears to promote healthy lifestyles and body positive ideals, its actual content largely revolves around the thin body ideal and fails to genuinely represent diverse body types [20]. Moreover, its emphasis on exercise and healthy eating is often directed toward enhancing appearance rather than prioritizing health itself [21]. This indicates that fitspiration does not truly align with the core tenet of body functionality theory, which emphasizes what the body can do rather than how it looks. Instead, it uses the language of functionality to reinforce appearance-focused norms, contradicting the principles of the DTE, which advocates for building positive body image through embodied physical experience [18]. In addition, fitspiration content frequently features sexualized portrayals of women through tight or revealing athletic wear and posed imagery [22], reinforcing objectifying views of the female body. Over time, such appearance-oriented content may lead to greater internalization of thin-ideal norms and increased self-objectification among women, thereby trapping them in a cycle of appearance-related anxiety [23,24].

To date, most experimental findings have confirmed these concerns. Studies indicate that fitspiration and thinspiration have very similar content, which concentrates on skinny and toned bodies [20,25,26]. Jeronimo and Carraca [16] published a systematic review including 20 studies in recent years regarding the effects of fitspiration content on body image, the result suggests that viewing fitspiration content always relates to greater levels of body dissatisfaction, negative moods, and appearance comparison tendencies like thinspiration does. Moreover, Russell [27] suggests that fitspiration content is not only as harmful as thinspiration content with regard to body image dissatisfaction and self-esteem, but may be more harmful than thinspiration with regard to inducing negative moods. Meanwhile, Dignard and Jarry [26] suggest that the negative impact of fitspiration cannot be offset by positive body images in the same manner as thinspiration, which suggests that fitspiration may be more harmful than thinspiration, especially among young women. These studies provide evidence that viewing fitspiration content is significantly associated with negative body perceptions, and this negative impact may be greater than that of thinspiration.

### The rationale for the study

From a review of past experiments, this research paper found that most studies were focused on Western samples, particularly young Caucasian women. However, the beauty standards of East Asian societies differ significantly from those in Western countries [28]. Unlike South or Central Asian countries, East Asian countries have experienced less colonial history [29]. As a result, local beauty standards are not entirely dependent on Eurocentric ideals but are more influenced by traditional culture and other East Asian countries [29]. For example, traditional Chinese aesthetics, largely viewed through a patriarchal lens, idealise the image of a delicate, frail Oriental women, emphasising slender and petite physiques [30]. Additionally, Japan’s Kawaii [cute] culture instills the belief that “women must be kawaii (cute)” from a young age [31] and, as a part of J-pop, has influenced beauty standards in other Asian countries [32]. Ultimately, East Asian beauty standards tend to portray women as youthful and innocent [29]. Therefore, while both East Asian and Western beauty standards emphasize “youthful” and “thin” ideals, they fundamentally differ in that East Asia’s interpretation of “youthful” and “thin” is more focused on conveying cuteness and innocence [29]. Due to cultural differences, societal beauty ideals in East Asian countries, including China, often diverge from those in Western societies [26]. Individuals from different ethnic and cultural backgrounds may respond to fitspiration through distinct psychological mechanisms [26]. In particular, East Asian women may prioritize thinness over functionality when exposed to fitspiration content, reflecting cultural norms that emphasize slenderness as a key aspect of attractiveness [33]. This highlights the need for more cross-cultural studies [34].

Further, studies have shown that Asian media tends to place a stronger emphasis on appearance than Western media, and that Asian women are more strongly influenced by media images from their own cultural context [35]. Given the above background, the present study focuses on mainland China and aims to explore Chinese women’s responses to fitspiration images from a culturally informed perspective.

To date, research on this topic in mainland China is limited. One study by Hong Kong scholars examined fitspiration’s impact on sexual minorities, finding associations with body surveillance and shame [36]. However, the sample size was limited to sexual minorities and women were not the primary focus of the research. Another qualitative study by Lee [37] analyzed Chinese fitspiration content and its gendered effects. One of the interesting findings was that men showed greater anxiety when dealing with fitspiration, while women showed admiration, which contrasts with previous studies conducted on Western samples [16,37]. While this study yields insightful and novel findings on the effects of fitspiration in the Chinese context, its conclusions are limited by its small sample size of only 20 participants [37]. Therefore, this study aims to increase the sample size and refine the previous findings through quantitative methods.

### The present study

The main objective of this study is to examine the impact of fitspiration on the body image perception of young Chinese women. Based on the objective, two research questions were made:

RQ1: To what extent does exposure to fitspiration imagery influence body image among young Chinese women?

RQ2: In what ways does the effect of fitspiration image exposure on body image differ from that of thinspiration image exposure among young Chinese women?

To answer the research questions and achieve the objective of the present study, the experiment assessed participants’ psychological indicators of body image (i.e., state body appreciation and body functionality appreciation level) before and after viewing fitspiration images, thinspiration images, and scenery images (as a control set). In the experiment, different visual stimuli served as the independent variables, while post-exposure state body appreciation and functionality appreciation of each visual set served as the dependent variables. Based on the findings of past studies, four hypotheses were made:

H1: Viewing fitspiration images will negatively influence body appreciation among young Chinese women.

H2: Viewing fitspiration images will lead to lower body appreciation compared to viewing thinspiration images among young Chinese women.

H3: Viewing fitspiration images will negatively influence functionality appreciation among young Chinese women.

H4: Viewing fitspiration images will lead to lower functionality appreciation compared to viewing thinspiration images among young Chinese women.

## Methods

### Participants

The study employed a multistage recruitment strategy targeting female undergraduate students aged 18–26 years from three universities in mainland China (one public, one private, and one vocational institution) to ensure diversity in educational backgrounds. A total of 432 participants were successfully recruited, yielding a response rate of 73%. The final sample (after exclusion, n = 414) had a mean age of 20.26 years (*SD* = 1.88) and an average BMI of 20.56 (*SD* = 3.86). All participants were Chinese citizens who self-identified as Asian Chinese.

Participant recruitment was conducted via institutional email invitations, which included a study information sheet and survey link, followed by two reminders at one-week intervals. Eligible candidates were identified through university enrollment records and randomly selected using an SPSS-generated random number sequence. Eligibility was confirmed through (a) demographic self-reports (age, gender, ethnicity) and (b) embedded attention-check items within the survey instrument. Any initially selected participants who failed to meet eligibility criteria were excluded and replaced using the same randomization procedures.

Data collection was carried out using the Wenjuanxing (WJX.cn) online survey platform, which offered real-time tracking of response status, IP address verification, timestamp monitoring, and automatic detection of incomplete submissions. The system maintained 100% sensitivity in identifying invalid or partial responses. No protocol violations or technical issues were recorded during the data collection period.

### Materials

#### Independent variables: visual stimulus.

The visual stimulus was used as independent variables in the experiment. They were split into three categories: (a) thinspiration images; (b) fitspiration images; and (c) scenery images (control group). Xiaohongshu is a leading appearance-based social media platform in China, with a predominantly young female user base [38]. It has been criticized for reinforcing unrealistic beauty standards through curated user-generated content, often featuring slim figures, flawless skin, and facial symmetry enhanced by photo editing [39,40]. Its algorithmic design promotes idealized images through features such as the tagging system and the Explore page, which prioritizes aesthetically appealing content [41]. These mechanisms foster upward appearance comparisons and body dissatisfaction [40]. Therefore, images used in this study were selected from Xiaohongshu to enhance ecological validity.

To ensure the accuracy of model image selections, this study followed the content criteria for thinspiration and fitspiration images outlined by Boepple and Thompson [20] and Lee [37]. Thinspiration images featured content related to weight or fat loss, praise for thinness, and thin body poses, while fitspiration images depicted bodies with visible muscle definition and athletic activity intended to inspire fitness. In addition, to reflect real social media usage, all images were selected from accounts with a large following or a high view count. In line with Williamson and Karazsia [42], the models’ faces were blurred to prevent bias based on facial appearance or recognition, which could influence the participants’ responses. This precaution helped eliminate potential bias from knowing the models. Moreover, models’ outfits were standardised to ensure consistency. For fitspiration images, models wore yoga outfits exposing their abdomen and body lines, while thinspiration images featured models in clothing that revealed a certain amount of skin (e.g., bikini).

Prior to the main experiment, a pilot evaluation was conducted to pretest and finalize the model image stimuli. A total of 10 young Chinese women who were not involved in the main study participated in the screening process. They evaluated 40 images (20 thinspiration and 20 fitspiration) sourced from Chinese social media platforms, using a Visual Analogue Scale (VAS) ranging from 0 (not at all) to 100 (very much). Participants were instructed to rate each image based on how well it reflected key characteristics of either thinspiration (e.g., thinness, appearance-focused content) or fitspiration (e.g., muscularity, functionality or fitness-oriented content). Based on the average VAS ratings across participants, the 10 highest-rated model images in each category were selected for use in the main study, resulting in a final model involved stimulus set of 20 images.

#### Dependent variable 1: post-exposure body appreciation.

The State Body Appreciation Scale-2 (SBAS-2) was used as a psychological measure to assess participants’ momentary body image levels before and after exposure to the visual stimulus. SBAS-2 was adapted by Homan [43] from the Body Appreciation Scale-2 (BAS-2) developed by Tylka and Wood-Barcalow [13]. BAS-2 has demonstrated strong construct validity in assessing positive body image perceptions [44]. A study that thoroughly assessed the measurement invariance of the BAS-2 across 65 countries, in 40 languages, which covered multiple gender identities, and age groups, confirmed that full scalar invariance was consistently upheld across these varied contexts [45]. Moreover, another study evaluating the Mandarin Chinese version of the BAS-2 in mainland China demonstrated strong construct validity, revealing positive correlations between body appreciation and self-esteem, self-compassion, and body satisfaction, as well as negative correlations with the Body Mass Index (BMI), weight discrepancy, negative effects, and body surveillance [46]. SBAS-2 modifies the BAS-2 by adding “right now” or “at this moment” to capture transient moods [43]. It retains a one-factor structure with strong validity and consistency, which is suited for assessing dynamic body appreciation [43]. Therefore, the Chinese version of SBAS-2 is suitable for use in the present study to assess the at-the-moment response of young Chinese women following their exposure to fitspiration images.

Participants rated ten items from the Chinese version of SBAS-2 [43,47] both before and after viewing visual stimuli (see S1 Appendix). The items included statements such as “Right now, I respect my body,” “At this moment, I feel good about my body,” and “Right now, I appreciate my body’s unique characteristics”. A five-point Likert Scale was used for participants to respond to each statement by selecting an option that best represented their current feelings: from 1 (strongly disagree) to 5 (strongly agree). Higher scores indicated a greater body appreciation. In the present study, the Cronbach’s Alpha score is 0.954, which indicates that the scale has good internal consistency.

#### Dependent variable 2: post-exposure functionality appreciation.

The Functionality Appreciation Scale (FAS), originally developed by Alleva, Tylka [48], is designed to assess the extent to which individuals value and appreciate the functions their bodies are capable of performing. Emphasizing body functionality has been recognized as an effective strategy for addressing negative body image and promoting body positivity, as it helps reduce appearance-related concerns, lowers the risk of eating disorders, and enhances psychological well-being [48]. Prior research has demonstrated a positive association between high functionality appreciation and positive body image, thereby supporting the use of the FAS to evaluate body image variations in the present study [49].

The Chinese version of the FAS (C-FAS) has shown excellent psychometric properties in previous studies conducted in China, with Cronbach’s alpha values ranging from 0.91 to 0.97 and strong construct validity [50]. Thus, its application in the current research context is both appropriate and psychometrically sound.

For this study, seven items from the C-FAS [50] were measured to assess participants’ appreciation of body functionality before and after exposure to the experimental stimuli (see S1 Appendix). Participants responded using a 5-point Likert scale ranging from 1 (strongly disagree) to 5 (strongly agree), with higher scores indicating greater functionality appreciation. In the present study, the adapted scale exhibited excellent internal consistency, with a Cronbach’s alpha of 0.953.

### Covariate variables

To control for baseline individual differences, two covariate variables were included: pre-exposure body appreciation and pre-exposure functionality appreciation. These variables were measured using the same instruments applied in the post-exposure phase, specifically the Chinese version of the State Body Appreciation Scale-2 (SBAS-2) and the Chinese version of the Functionality Appreciation Scale (C-FAS). Using identical measures at both time points ensured consistency in the assessment. The Cronbach’s alpha coefficients for the pre-exposure measures were α = 0.957 for SBAS-2 and α = 0.951 for FAS.

### Personal information

At the start of the questionnaire, participants were asked about their perceived sex and age to ensure they met the eligibility criteria. Additionally, questions on height and weight were included to determine Body Mass Index [BMI] ratings, which allowed for further sample characteristic descriptions.

### Attention check

An attention check was included in the questionnaire to ensure participants were engaged with the content. Specifically, participants were asked to select “Strongly Disagree” on one of the five Likert scale items. Responses that did not match this instruction were flagged for further review and excluded from the analysis to maintain the integrity of the data.

### Procedure

**Fig 1:**
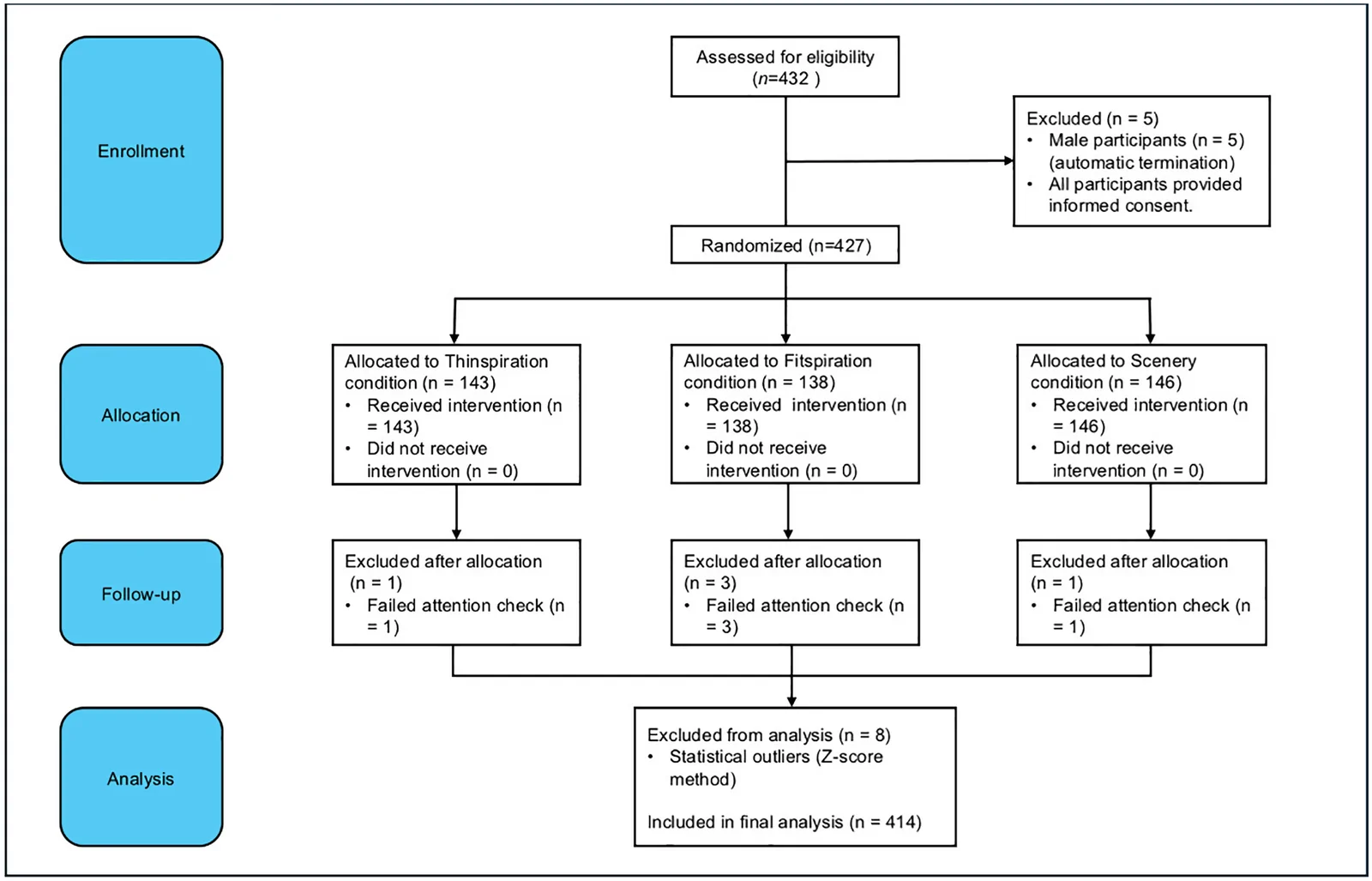
CONSORT-style Flow Diagram of the Experimental Procedure.

In accordance with the requirements of the University of Malaya Research Ethics Committee (UMREC), ethics approval was obtained prior to data collection (Approval No. UM.TNC2/UMREC_3512; contact email: umrec@um.edu.my). The recruitment and data collection period subsequently took place from 13 August 2024–15 October 2024. A between-subjects experimental design was employed, wherein participants were randomly assigned to different stimulus sets to avoid potential carryover effects and ensure the independence of observations, which is the most commonly used design as seen in related studies [11,51,52]. The whole process is presented in a CONSORT-style flow diagram (Fig 1). Specifically, participants first joined a WeChat group and received a questionnaire link via Wenjuanxing (a professional questionnaire platform in China). Informed consent was obtained before starting. Specifically, a consent statement was displayed in a pop-up window immediately upon opening the online questionnaire. Participants were required to confirm their agreement before proceeding to the experiment. Only those who provided informed consent were able to continue and complete the questionnaire. Subsequently, initial personal information, including perceived sex, age, weight, and height (for BMI calculation), was collected to ensure participant eligibility. The gender field was set to automatically end the questionnaire if “male” was selected. Then, participants first completed a pre-exposure test measuring SBAS-2 and FAS using Likert scales. After that, they were randomly assigned to one of three stimulus sets: (a) thinspiration images; (b) fitspiration images; or (c) scenery images. Each set contained ten images. The survey system was programmed to enforce a minimum viewing time of 30 seconds per image before participants could proceed. After the exposure, participants were asked to finish the post-exposure test measuring SBAS-2 and FAS using Likert scales. An attention check question was displayed at the end of the questionnaire. There was almost no break between the pre-exposure and post-exposure for participants. After completing all questions, a thank-you message was displayed. The test took approximately eight minutes in total to complete, and no personally identifiable information was collected from participants.

### Statistical methods

#### Data cleaning.

Since the experimental data were collected and stored via an online platform, there were no missing values. Upon exporting the dataset to Excel, the researcher conducted data cleaning in two stages. First, responses that clearly did not meet the inclusion criteria for the experimental sample were excluded. Specifically, data from participants who did not meet the demographic requirements (e.g., male participants, n = 5) were removed. Additionally, participants who failed the attention check were excluded. The attention check item required participants to select “Strongly Disagree,” and those who selected any other option were considered inattentive and subsequently removed (n = 5). Second, the remaining data were screened for outliers using the Z-score method. The Z-score for each participant’s score was computed by subtracting the sample mean from the individual value and dividing the result by the sample standard deviation. Z-scores were calculated separately for Pre-BAS, Pre-FAS, Post-BAS, and Post-FAS scores. Any data point with a Z-score greater than 3 was identified as an outlier and removed from the dataset. This procedure led to the exclusion of an additional 8 cases, resulting in a final sample of 414 valid responses.

The *a priori* power analysis was conducted by using G*Power [53]. Analysis of covariance (ANCOVA) was selected for the analysis, using a medium effect size of 0.25 based on previous studies. A power of 0.95 was established to ensure adequate statistical power. With three groups and two covariates, the software indicated that a minimum of 400 participants was required. Ultimately, our final sample of 414 participants exceeded this requirement, demonstrating the effectiveness of the data analysis.

### Data analysis

All statistical analyses were performed using IBM SPSS Statistics 27.0 (IBM Corp., Armonk, NY, USA). Descriptive statistics were first computed to summarize participants’ demographic characteristics, including age, height, weight, and body mass index (BMI). BMI was calculated using the standard formula (weight in kilograms divided by height in meters squared), and participants were categorized according to the BMI standards for Chinese populations [54]: underweight (BMI < 18.5), normal weight (18.5 ≤ BMI < 24), and overweight (BMI ≥ 24). The number of people and percentages for each BMI category were reported to describe the distribution of body composition within the sample.

Group-level descriptive statistics, including the means and standard deviations of pre- and post-exposure scores on the State Body Appreciation Scale-2 (SBAS-2) and the Functionality Appreciation Scale (FAS), were computed for each image exposure condition (thinspiration, fitspiration, and scenery). In addition to raw means, covariate-adjusted means (estimated marginal means) and corresponding standard errors were calculated based on ANCOVA results to account for baseline individual differences. These adjusted values provided a more accurate estimation of group differences after controlling for pre-test scores.

To evaluate the effect of image exposure on body appreciation and functionality appreciation, two separate one-way ANCOVAs were conducted. The independent variable was the type of image viewed, and the dependent variables were the post-exposure scores on SBAS-2 and FAS. The corresponding pre-exposure scores were entered as covariates to statistically control for individual baseline differences.

Before conducting the ANCOVAs, the assumptions of the analysis were carefully examined and satisfied. The assumption of normality was assessed using the Shapiro–Wilk test and supported by visual inspection of histograms and Q–Q plots. Homogeneity of variances was confirmed via Levene’s test, which indicated no significant differences in variance across groups (*p* > .05). Furthermore, the assumption of homogeneity of regression slopes was tested by ensuring that there was no significant interaction between the covariate and the independent variable. Pearson correlations also confirmed that the covariates (pre-test scores) were significantly associated with their respective dependent variables (post-test scores), justifying their inclusion in the models.

The significance level was set at *p* < .05 for all analyses. Effect sizes were reported using partial eta squared (η²) to indicate the magnitude of the observed effects, with values of  .01,  .06, and  .14 generally interpreted as small, medium, and large effects, respectively. Post hoc pairwise comparisons with Bonferroni correction were conducted where appropriate to explore group differences.

## Results

### Sample characteristics

#### Sample’s basic demographic characteristics.

Table 1 shows the participants’ basic demographic characteristics. All participants were Asian Chinese with a mean age of 20.26 years (*SD* = 1.88) and a mean BMI of 20.56 (*SD* = 3.86). Based on BMI classification, 28.02% were underweight (BMI < 18.5), 61.35% were of normal weight (BMI 18.5–24.0), and 10.63% were classified as overweight (BMI ≥ 24.0). This distribution reflects common body weight patterns among young adult women in contemporary urban China, where thinness is often socially idealized.

**Table 1:** Participants’ Demographic Characteristics.

Characteristic	Value
Ethnicity	100% Asian Chinese
Mean Age (*SD*)	20.26 (1.88) years
Mean BMI (*SD*)	20.56 (3.86)
**BMI Category**	**n (% of total)**
Underweight (BMI < 18.5)	116 (28.02%)
Normal Weight (BMI 18.5–24.0)	254 (61.35%)
Overweight (BMI ≥ 24.0)	44 (10.63%)

### Descriptive statistics of the sample

Descriptive statistics (Table 2) indicated that all three groups were comparable at baseline in both state body appreciation (SBAS) and functionality appreciation (FAS). After image exposure, adjusted post-test scores showed that participants in the scenery condition reported the highest levels of both body appreciation (*M* = 39.13, *SE* = 0.29) and functionality appreciation (*M* = 32.06, *SE* = 0.10), followed by the fitspiration group (SBAS: *M* = 36.99, *SE* = 0.30; FAS: *M* = 29.47, *SE* = 0.10), and the thinspiration group reported the lowest levels (SBAS: *M* = 32.56, *SE* = 0.29; FAS: *M* = 27.62, *SE* = 0.10).

**Table 2:** Descriptive Statistics Across the Conditions.

	Thinspirationn=140 *M*(*SD*)	Fitspirationn=130 *M* (*SD*)	Sceneryn=144 *M* (*SD*)
**SBAS**			
Pre	37.67 (7.38)	40.36 (8.29)	36.67 (7.19)
Post (Raw)	32.06 (8.21)	39.22 (9.67)	37.61 (7.44)
Post (CV adjusted)	32.56^a^ (*SE*=0.29)	36.99^a^ (*SE*=0.30)	39.13^a^ (*SE*=0.29)
**FAS**			
Pre	30.69 (3.93)	31.06 (4.66)	28.98 (5.57)
Post (Raw)	28.07 (3.91)	30.26 (4.66)	30.90 (5.24)
Post (CV adjusted)	27.62^b^ (*SE*=0.10)	29.47^b^ (*SE*=0.10)	32.06^b^ (*SE*=0.10)

CV=covariate; *SE*=standard error.

a b Evaluate the covariate in the model using the following value: Pre-BAS = 38.17, Pre-FAS = 30.21.

### Results of ANCOVA

#### State body appreciation.

A one-way ANCOVA was conducted to examine the effects of image type (fitspiration, thinspiration, scenery) on post-test state body appreciation (Post-SBAS) among young Chinese women, controlling for pre-test SBAS scores (Table 3). The overall model was significant, F (3, 410) = 806.76, p < .001, with a large effect size (partial η² = .855), indicating a large proportion of variance in post-test body appreciation is associated with the model. The covariate, Pre-SBAS, was also a significant predictor, F (1, 410) = 2090.29, p < .001, partial η² = .836. A significant main effect of image type was found, F (2, 410) = 135.25, *p* < .001, partial η² = .398, indicating that the type of image exposure had a substantial influence on participants’ body appreciation levels.

**Table 3:** Tests of Between-Subjects Effects for SBAS-2.

Source	Type III Sum of Squares	df	Mean Square	F	Sig.	Partial Eta Squared
Corrected Model	28392.964	3	9464.321	806.757	.000	.855
Intercept	97.881	1	97.881	8.344	.004	.020
Pre-SBAS	24521.896	1	24521.896	2090.294	.000	.836
Image Type	3173.400	2	1586.700	135.253	.000	.398
Error	4809.838	410	11.731			
Total	576826.000	414				
Corrected Total	33202.802	413				

R^2^ =  .855 (Adjusted R^2^ =  .854). Dependent Variable: Post-SBAS.

Bonferroni-adjusted pairwise comparisons (Table 4) indicated that participants in the fitspiration condition reported significantly lower Post-SBAS scores than those in the scenery condition (mean difference = –2.145, 95% CI [–3.160, –1.129], *p <* .001). In contrast, Post-SBAS scores in the fitspiration condition were significantly higher than those in the thinspiration condition (mean difference = 4.429, 95% CI [3.416, 5.442], *p <* .001). These findings supported H1 but did not support H2.

**Table 4:** Bonferroni-Adjusted Pairwise Comparisons for Post-SBAS.

[I] Image Type	[J] Image Type	Mean Difference [I–J]	Std. Error	Sig. (*p*)	95% Confidence Interval
Fitspiration	Scenery	–2.145*	0.422	.000	[–3.160, –1.129]
	Thinspiration	4.429*	0.421	.000	[3.416, 5.442]
Thinspiration	Fitspiration	–4.429*	0.421	.000	[–5.442, –3.416]
	Scenery	–6.573*	0.407	.000	[–7.552, –5.595]
Scenery	Fitspiration	2.145*	0.422	.000	[1.129, 3.160]
	Thinspiration	6.573*	0.407	.000	[5.595, 7.552]

Based on estimated marginal means. Adjustment for multiple comparisons: Bonferroni. *The mean difference is significant at the 0.05 level.

### Functionality appreciation

A one-way ANCOVA was conducted to examine the effects of image type (fitspiration, thinspiration, scenery) on post-test functionality appreciation (Post-FAS) among young Chinese women, controlling for pre-test FAS scores (Table 5). The overall model was significant, F (3, 410) = 2159.52, *p <* .001, with a large effect size (partial η² = .940). The covariate, Pre-FAS, was also a significant predictor, F (1, 410) = 6027.77, *p <* .001, partial η² = .936. A significant main effect of image type was found, F (2, 410) = 500.93, *p <* .001, partial η² = .710, indicating that the type of image exposure had a substantial influence on participants’ functionality appreciation levels.

**Table 5:** Tests of Between-Subjects Effects for FAS.

Source	Type III Sum of Squares	df	Mean Square	F	Sig. (*p*)	Partial Eta Squared
Corrected Model	8906.715	3	2968.905	2159.521	.000	.940
Intercept	17.281	1	17.281	12.570	.000	.030
Image Type	1377.367	2	688.683	500.934	.000	.710
Pre-FAS	8286.970	1	8286.970	6027.773	.000	.936
Error	563.667	410	1.375			
Total	375687.554	414				
Corrected Total	9470.382	413				

R^2^ =  .940 (Adjusted R^2^ =  .940)

Bonferroni-adjusted pairwise comparisons (Table 6) showed that the fitspiration group reported significantly lower Post-FAS scores than the scenery group (mean difference = –2.592, 95% CI [–2.938, –2.246], *p <* .001). However, Post-FAS scores in the fitspiration condition were significantly higher than those in the thinspiration condition (mean difference = 1.846, 95% CI [1.502, 2.189], *p <* .001). Thus, H3 was supported, whereas H4 was not supported.

**Table 6:** Bonferroni-Adjusted Pairwise Comparisons for Post-FAS.

[I] Image Type	[J] Image Type	Mean Difference [I–J]	Std. Error	Sig. [p]	95% Confidence Interval
Fitspiration	Scenery	–2.592*	0.144	.000	[–2.938, –2.246]
	Thinspiration	1.846*	0.143	.000	[1.502, 2.189]
Thinspiration	Fitspiration	–1.846*	0.143	.000	[–2.189, –1.502]
	Scenery	–4.437*	0.141	.000	[–4.776, –4.099]
Scenery	Fitspiration	2.592*	0.144	.000	[2.246, 2.938]
	Thinspiration	4.437*	0.141	.000	[4.099, 4.776]

Based on estimated marginal means. Adjustment for multiple comparisons: Bonferroni. *The mean difference is significant at the 0.05 level.

## Discussion

This study was among the first to experimentally examine the impact of viewing fitspiration images on social media on body and functionality appreciation in Chinese women. Based on the results, H1 and H3 were supported, while H2 and H4 were rejected. Overall, the present study made two key findings. Firstly, that fitspiration images have a significant association with lower body and functionality appreciation among young Chinese women. Secondly, compared to fitspiration, thinspiration tends to have a more detrimental effect on body and functionality appreciation for Chinese women. The following content discusses the key findings.

### Negative impact of fitspiration

The study found that viewing fitspiration images can significantly decrease young Chinese women’s body and functionality appreciation, which may be linked to negative body image perceptions. This finding is consistent with most Western findings. According to a systematic review by Jeronimo and Carraca [16], fitspiration content has a proven significant association with negative moods, body dissatisfaction, and self-objectification. Based on previous research, two main factors contribute to these results. First, fitspiration images feature unattainable idealised bodies, which create a gap between the ideal and reality for average women, leading to feelings of defeat and generating negative emotions after viewing such images [55]. Second, viewing fitspiration images tends to promote the internalisation of the muscular ideal, which, in turn, reinforces women’s tendency toward appearance-based comparisons [16]. As a result, when women see images of thin and muscular bodies on social media, they are more likely to engage in upward appearance comparisons, ultimately leading to a negative body image [23].

Although there is no other direct research in mainland China examining the impact of fitspiration on a women’s body image, related studies support this perspective. For instance, Ding and Xu [56] found that sociocultural pressure moderated the relationship between appearance-based social comparison and anxiety by highlighting the link between social media and social comparison. Similarly, Lin, Lu [57] noted that Chinese society is increasingly emphasising a muscular ideal for men, and when this ideal is embodied by male idols, their female followers may also adopt a preference for muscular aesthetics. In other words, as fitspiration reflects societal ideals, the muscularity-driven sociocultural pressure can lead women to engage in upward appearance comparisons, resulting in heightened body anxiety. Additionally, Zhang, Zhu [40] indicated that the negative effects of viewing idealized images were moderated by an individuals’ self-discrepancy between personal ideals and their own bodies. This suggests that when there is a significant gap between one’s own appearance and the ideal image presented by fitspiration, the negative impact on body image becomes more pronounced. These insights reveal that in both Eastern and Western cultural contexts, fitspiration can reinforce a negative body image. The primary reason lies in fitspiration’s tendency to foster upward appearance comparisons by internalising and displaying unattainable ideals. Therefore, Chinese social media platforms must also recognise the negative impact of fitspiration and develop appropriate media literacy initiatives to address its effects.

### Fitspiration was less detrimental than thinspiration

The results showed that exposure to fitspiration images led to significantly higher levels of body and functionality appreciation compared to thinspiration images, despite both being less effective than neutral imagery. This finding is consistent with some previous studies. For instance, Griffiths and Stefanovski [25] found that both thinspiration and fitspiration negatively affect body image and emotional well-being, with thinspiration showing more consistent negative effects. Similarly, Griffiths, Castle [58] found that exposure to thinspiration images was associated with more severe eating disorder symptoms, demonstrating stronger links to symptom severity compared to fitspiration content. This can be attributed to thinspiration’s emphasis on extreme thinness, while fitspiration, though idealising muscular bodies, initially promotes a positive message of health and exercise [21,59]. These findings indicate that, despite its idealized nature, fitspiration content exerts a comparatively less detrimental impact on body and functionality appreciation than thinspiration content.

Conversely, other studies argue that fitspiration may have a more detrimental impact than thinspiration. Dignard [60] highlighted that both fitspiration and thinspiration can lead to body dissatisfaction through appearance comparisons. However, fitspiration viewers reported higher appearance comparisons, suggesting that fitspiration could induce more upward comparisons and ultimately lower body satisfaction. This was further supported by Dignard and Jarry [26], who found that fitspiration was correlated to lower body satisfaction compared with thinspiration, as thinspiration viewers with a positive body image might experience less appearance comparison. Moreover, Russell [61] argued that fitspiration can cause more severe negative moods than thinspiration, highlighting fitspiration’s potential to worsen negative body image perceptions.

Two main factors explain why this study found that fitspiration has less detrimental effects than thinspiration. First, as indicated in other Western studies, fitspiration’s messaging is less extreme and sometimes promotes a positive lifestyle [21,59]. Second, China’s unique cultural context influences how Chinese women perceive fitspiration. According to studies favouring the idea that fitspiration has more severe negative effects, appearance comparisons and sociocultural ideals play a significant role in body dissatisfaction [4]. In societies where thinspiration aligns more closely with societal ideals, individuals are more likely to engage in upward comparisons with thinspiration images; conversely, fitspiration may elicit upward comparisons in contexts where it resonates less with traditional ideals. As a Western import, the athletic-focused fitspiration ideal has weaker roots in China [37]. In contrast, thinspiration aligns with China’s traditional aesthetic, which prizes thinness [23,30].

Additionally, the slower development of the body positivity movement in China contrasts with Western societies, where awareness of fitspiration’s potential to harm is more prevalent [62]. Only in late 2020 did body positivity discussions gain traction on Chinese social media [38]. Chinese women still primarily focus on resisting thinspiration, while fitspiration is often praised or accepted [37,38]. Chinese women may interpret fitspiration’s display of muscle tone as representing “female strength,” aligning with recent feminist narratives in China. This might lead to a more positive perception of fitspiration, reducing the impact of the negative side.

Furthermore, China’s long-standing patriarchal culture has ingrained self-objectification in women, who may subconsciously strive to meet male-centric aesthetic standards. According to objectification theory, women internalise self-objectification due to pervasive objectifying experiences [24]. China’s deeply rooted patriarchy has historically shaped this mindset, which persists despite recent feminist efforts [63]. Unlike Western ideals, Chinese male preferences do not emphasise muscularity in women but are aligned with a cute and innocent image, which conveys submissiveness [63,64]. Consequently, fitspiration’s muscular aesthetic does not fulfil this objectified ideal as strongly as thinspiration, resulting in fewer upward comparisons. This perspective is echoed across East Asian societies. For example, Cataldo, Burkauskas [65] noted that Japanese women reported lower body anxiety from fitspiration than their Western counterparts, likely due to similar aesthetic preferences (such as “Kawaii”) influenced by patriarchal values [66]. Therefore, unlike Western research, which attributes the negative body image effects of fitspiration to the internalisation of muscular ideals that lead to upward comparisons [21,67], the negative impact of fitspiration on Chinese and Japanese women is not due to the athletic-oriented images it portrays but rather due to the thinness ideal embedded within them. This difference highlights how cultural standards influence the way women react to fitspiration, with thinness being a more significant factor than muscularity in shaping body dissatisfaction.

In summary, China’s nascent body positivity movement means Chinese women may not fully recognize fitspiration’s potential risks. Chinese women’s beauty ideals, shaped by patriarchal influence, continue to prioritise non-muscular thinness, leading to a perception that the impact of fitspiration’s upward comparison is milder than thinspiration’s. However, due to its underlying thinness ideals, fitspiration still has the potential to impact young Chinese women negatively. As body positivity and feminist movements grow in China, the awareness of fitspiration’s potential pitfalls may increase. Hence, while fitspiration may currently seem less harmful than thinspiration, media professionals and social media platforms should remain vigilant about its potential risks and implement proactive measures for prevention or mitigation. For example, although the body positivity movement has gained widespread recognition, some researchers have noted that it may, in certain contexts, inadvertently reinforce appearance-related pressures, promote extreme attitudes, or be commercialized [68]. To address these potential issues, the concept of “body neutrality” has been proposed, emphasizing a more balanced perspective that focuses on bodily functionality and health rather than solely on positive appearance attitudes, thus providing opportunities for future research and practice to promote healthier body image strategies [12].

### Limitation

Several limitations of this study should be acknowledged. First, although the sample included women aged 18–26, the mean age was 20.26 years (*SD* = 1.88), suggesting that participants were predominantly at the younger end of the range. This may limit the generalizability of the findings to older individuals within the same age bracket. Moreover, all participants were recruited from universities, which may further narrow the representativeness of the sample. University students may differ from their non-student peers in terms of lifestyle, media use, and body image concerns. Future research should consider recruiting participants from a broader range of social contexts, such as workplaces, community organizations, or online platforms, to capture more diverse perspectives among young adult women.

Second, the study employed a cross-sectional experimental design with only immediate pre- and post-exposure assessments. While this enabled the detection of short-term effects, it did not allow for the evaluation of long-term or cumulative impacts. Given the repetitive nature of social media use, future research should consider longitudinal designs to assess whether and how media-induced changes in body or functionality appreciation persist or evolve over time.

Third, although the study controlled for several key factors, there may still be uncontrolled variables that could have influenced the results, such as participants’ recent exposure to social media, mood states, or individual differences in baseline body satisfaction. Meanwhile, while the study focused on body and functionality appreciation as key outcomes, it did not include direct measures of negative body image, such as body dissatisfaction, appearance anxiety, or disordered eating attitudes. Although low body and functionality appreciation may indicate body image challenges, it is not equivalent to clinically significant distress or dysfunction. Including a broader range of body image constructs in future studies would provide a more comprehensive understanding of the psychological effects of exposure to idealized images.

## Conclusion

This study found that both fitspiration and thinspiration images negatively affected young Chinese women’s body and functionality appreciation, with thinspiration being the most harmful. While fitspiration appeared less detrimental, it still promoted thinness ideals and led to reduced body and functionality appreciation compared to neutral imagery.

In China’s early-stage body positivity context, fitspiration’s risks may be under-recognized due to prevailing beauty standards that prioritize slenderness over muscularity. Unlike Western contexts where toned and muscular physiques are often emphasized, Chinese beauty ideals place less value on visible muscle definition. As a result, fitspiration images may evoke weaker upward social comparison than thinspiration images, as the depicted body ideals are less aligned with dominant appearance norms. This reduced comparative pressure may partially explain why fitspiration appears less harmful, although it still reinforces appearance-focused evaluation. As feminist and body-positive awareness grows, future research should pay greater attention to the nuanced impact of fitspiration from a culturally specific Chinese perspective.

## Supporting information

S1 Appendix CN/EN Scale Items Used in The Experiment. (DOCX)
