# Supplementary material for: Rethinking fitspiration in social media: Experimental insights from young Chinese women
Source: PLoS One. 2026 Sep 3;21(9):e0357611. doi: 10.1371/journal.pone.0357611 (PMC13541199; doi:10.1371/journal.pone.0357611)
Supplement: Supplementary file 1 [file pone.0357611.s001.docx]

**CN/EN Scale Items Used in The Experiment**

**SBAS-2:**
1. 现在，我尊重自己的身体/Right now, I respect my body

2. 此时此刻，我自觉的我的身材还算不错/At this moment, I feel good about my body.

3. 现在，我觉得我的身材有些还算不错的地方。/Right now, I feel that my body has at least some good qualities.

4. 此时此刻，我对我的身材抱有正面的态度。/At this moment, I take a positive attitude towards my body.

5. 现在，我关注我的身体所需。/Right now, I am attentive to my body’s needs

6. 此时此刻，我喜欢我的身体。/At this moment, I feel love for my body.

7. 现在，我欣赏我自己所拥有独特和不同的体型。/Right now, I appreciate the different and unique characteristics of my body.

8. 此时此刻，我用正面的态度来对待我的身体。例如，抬高头和对人微笑。/At this moment, my behaviour reveals my positive attitude toward my body; for example, I hold my head high and smile.

9. 现在，我安逸于自己的体型。/Right now, I am comfortable in my body.

10. 此时此刻，虽然我不像媒体的人物那样吸引，但我仍然觉得自己美丽。/At this moment, I feel like I am beautiful even if I am different from media images of attractive people (e.g., models, actresses/actors)

**FAS:**

1. 现在，我对自己身体的健康心存感激，尽管它并不总是像我希望的那样健康。/ Right now, I am grateful for the health of my body, even if it isn't always as healthy as I would like it to be.

2. 此时此刻，我感谢我的身体，感谢它所能做的一切。/ At this moment, I appreciate with my body for what it is capable of doing.

3. 现在，我感谢我的身体让我能够与他人交流和互动。/ Right now, I appreciate that my body allows me to communicate and interact with others.

4. 此时此刻，我承认并感激我的身体让我感觉良好和/或放松。/ At this moment, I acknowledge and appreciate when my body feels good and/or relaxed.

5. 现在，我感谢我的身体让我能够从事我喜欢或认为重要的活动。/Right now, I am grateful that my body enables me to engage in activities that I enjoy or find important.

6. 此时此刻，我觉得我的身体为我做了很多./ At this moment, I feel that my body does so much for me.

7. 现在,我尊重我的身体所发挥的功能/Right now, I respect my body for the functions that it performs
